# Digitizing chemical discovery with a Bayesian explorer for interpreting reactivity data

**DOI:** 10.1073/pnas.2220045120

**Published:** 2023-04-17

**Authors:** S. Hessam M. Mehr, Dario Caramelli, Leroy Cronin

**Affiliations:** ^a^School of Chemistry, University of Glasgow, Glasgow G12 8QQ, UK

**Keywords:** chemputing, Bayesian explorer, reactivity data

## Abstract

Interpreting the outcome of chemistry experiments consistently is slow and frequently introduces unwanted hidden bias. This difficulty limits the scale of collectable data and often leads to exclusion of negative results, which severely limits progress in the field. What is needed is a way to standardize the discovery process and accelerate the interpretation of high-dimensional data aided by the expert chemist’s intuition. We demonstrate a digital Oracle that interprets chemical reactivity using probability. By carrying out >500 reactions covering a large space and retaining both the positive and negative results, the Oracle was able to rediscover eight historically important reactions including the aldol condensation, Buchwald–Hartwig amination, Heck, Mannich, Sonogashira, Suzuki, Wittig, and Wittig–Horner reactions. This paradigm for decoding reactivity validates and formalizes the expert chemist’s experience and intuition, providing a quantitative criterion of discovery scalable to all available experimental data.

## Significance

Critical issues in automated chemistry discovery include cherry picking, disregarding negative data, and arbitrary interpretation of outcomes. Robotic data collection has accelerated experimentation but not addressed consistent interpretation. We introduce a Bayesian reasoning system that accounts for user bias, utilizes all data, and provides confidence values for deductions. Working with a robotic platform, it interprets experiment outcomes for chemists and designs new experiments, automating the process without hidden bias and quantifying discovery based on prior knowledge and observed data.

Across chemistry, discovering new chemical reactions and compounds is a time-consuming and labor-intensive process (1–3). Robotic chemistry platforms promise to accelerate discovery by performing experiments with unprecedented reproducibility and throughput (4–9), but in the absence of a mechanism to formulate experiments and interpret their results, automated systems can only reduce the manual burden (10). When equipped with online analytics and chromatography, these platforms have the hardware components necessary for chemical discovery in a closed loop by accumulating a body of knowledge from successive experiments (11). However, the chemical insight required to close the loop must still be supplied by a human expert, who is easily overwhelmed by the volume of information (12, 13), while also introducing implicit and unmeasurable bias to the discovery process (14–16). The result is that discovery efforts fail to communicate the assumptions and chain of reasoning leading to key findings (17). As chemical space is vast (18), it is critical to document the evolving constraints imposed on the experimental space—such as the choice of starting materials and process variables, for discovery to build on a planned experimental trajectory rather than pure serendipity (19, 20).

**Fig. 1.**
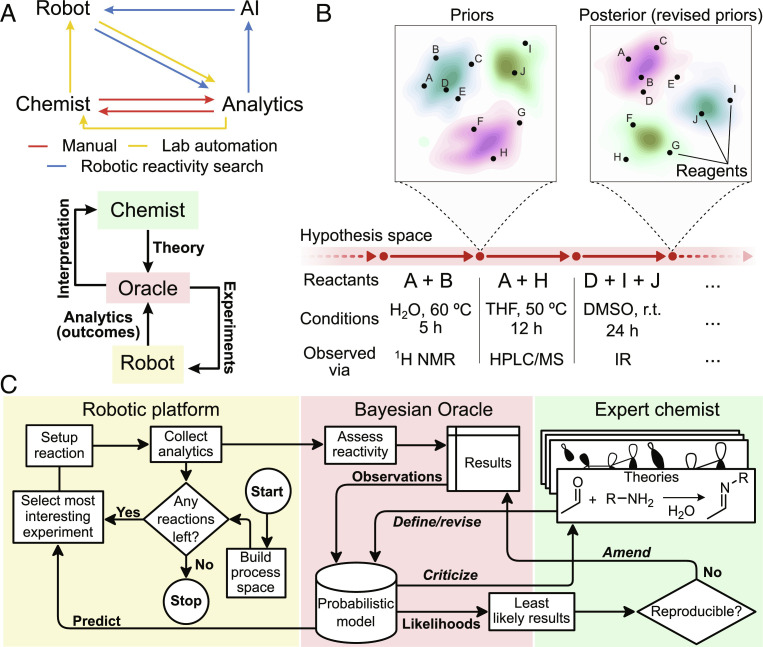
(*A*) Progress in discovery workflows. The most primitive form of automation simply replaces the human for labor-intensive operations. AI-powered robotic reactivity search using online analytics has recently become possible but normally operates as a black box without clear input or interpretability by the expert. True hybrid AI-human discovery relies on human intuition to enable generalizing interpreting and interrogating discoveries as well as the assumptions leading to them. (*B*) Abstract representation of probabilistic discovery. Expert knowledge expressed in terms of a quantitative probabilistic model corresponds to a single point in hypothesis space. Evaluating the current hypothesis and its remaining gaps, new experiments can be formulated—in this case, specified as reagents, conditions, and observation method—for execution automatically by the robot or manually if necessary. The outcome of each experiment (observation) updates the model’s priors, taking exploration a step forward in hypothesis space. (*C*) Integration of probabilistic interpretation into a closed-loop robotic discovery platform. The experimental platform iterates over the remaining experiments, picking the most “interesting” reaction as recommended by the Oracle in each iteration. Acquired data from the experiments are processed by the Oracle, resulting in a shortlist of surprising reactions to be further investigated by expert chemists in order to discover new compounds or amend existing theories. Actions involving interaction with the expert are shown in italic type.

Seeking to eliminate the dependence on human input, various machine learning models have been proposed to predict the outcome of chemical reactions (21–25). Such systems rely on deep neural networks or other regression models to predict experiment outcomes from the robot’s input, typically formulation and experimental process variables such as reaction temperature (26). This approach has shown promise for reaction optimization and replaced lengthy design of experiments (DOE) procedures in many cases, but progress so far has been limited to accelerating the search for reactivity irrespective of its source (27). Recent approaches to discovery seek to eliminate human bias to maximize the novelty of potential discoveries (28, 29), but any connection between the algorithm’s understanding of chemistry and human intuition necessarily introduces bias. Rather than eliminating or reducing bias, our aim was to maintain the grounding of discovery results in hypotheses, a powerful connection that has been explored in earlier work (30), while specifically eliminating hidden/implicit bias. To this end, we sought to couple an autonomous chemical system with an expert-defined digital model of chemical reactivity that makes all sources of human bias explicit and quantifiable, Fig. 1*A*.

We present a Bayesian Oracle that acts as an interpreter for robotic chemistry platforms. Within the Oracle, the chemist’s understanding of chemistry can be encoded as a probabilistic model connecting the reagents and process variables in each experiment to observed quantities, such as spectroscopic evidence of reactivity. The qualitative relationship between entities is captured by their connectivity within the probabilistic model. Additionally, the quantitative interdependence of observed and latent quantities is described using prior probability distributions that describe existing beliefs and are continuously refined as the robot attempts different experiments and uses online analytics to make observations (Fig. 1*B*). Bayes’ theorem provides a sound theoretical framework for realizing probabilistic models (31) amenable to high-performance numerical implementation using Markov chain Monte Carlo (MCMC) (32–34) as well as variational inference techniques (35, 36).

The Oracle interfaces with a robotic chemistry platform designed to perform combinatorial experiments from a set of starting materials and assess reactivity via a set of online analytical instruments. Using these data as observations, the probabilistic model can formulate a general concept of chemical reactivity for the reagents and answer queries relating to past and future experiments. Furthermore, by computing the likelihood of each experimental outcome, the system is able to assess the significance of the results seen so far and highlight experiments with surprising outcomes. The shortlist of unexpected reactivity can be utilized by an expert chemist for validation and, with the product isolated, formulation of new reactivity types and mechanisms. The expert can modify or refine their theory, instantly updating the workflow, Fig. 1*C*.

We initially validated our system in silico with the simulated discovery of two historical named reactions (Diels–Alder and Passerini reactions) before using it in conjunction with our robotic platform to acquire experimental data from a rich chemical space. Analyzing reaction outcomes via high-performance liquid chromatography (HPLC), nuclear magnetic resonance spectrometry (NMR), and mass spectrometry (MS) from a rich chemical space allowed us to simulate the discovery of nine historically important reactions. The data were processed to extract relevant reaction information, and the probabilistic model was able to independently interpret and assess the novelty of the outcomes corresponding to the named reactions. This experiment confirms that our probabilistic workflow can be used by chemists as a quantitative framework for assessing the significance and mechanistic consequences of new experimental findings.

## Probabilistic Model

Current attempts to automate discovery take an ad hoc approach to the problem of finding novelty (10, 37). The search in chemical space is often guided by predicted reactivity, but the desired outcome is often making a discovery, i.e., reactivity that appears unlikely according to previous knowledge. We sought to create a framework wherein expert chemists can describe their theories—including any bias from their training and experience—quantitatively as a probabilistic model. This quantitative description makes it possible to define discoveries formally in terms of the state of beliefs before and after a set of experiments.

**Fig. 2.**
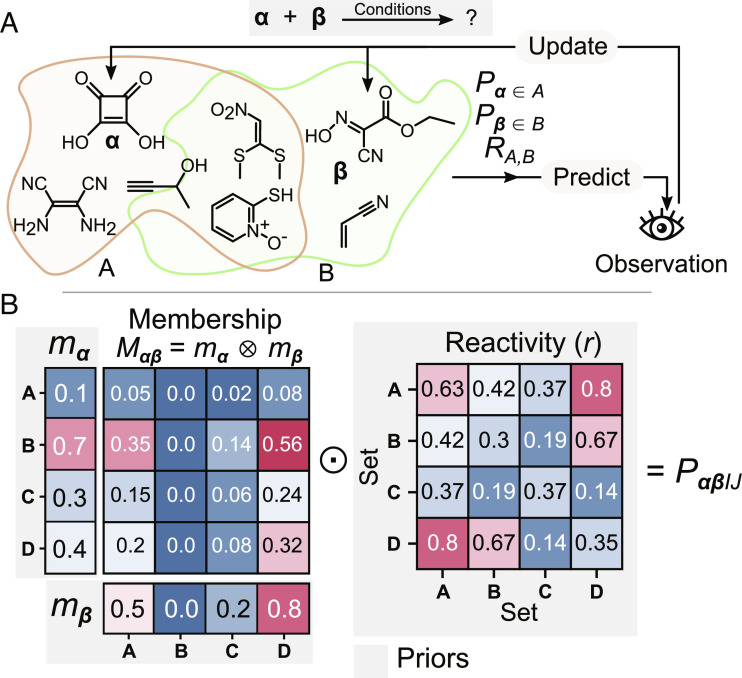
(*A*) Description of a simple probabilistic model used to validate our system. Compounds are hypothesized to possess varying degrees of a set of properties, with each property showing varying degrees of reactivity toward other properties. Reactivity observations are used to infer likely allocations of properties and mutual reactivities. (*B*) Combination of membership *M_αβ_* and reactivity R matrices for compounds α and β to yield the probability component matrix *P_αβIJ_*, which expresses the probability that α and β will react as a result of belonging to sets *I* and *J*, respectively. The ⊗ and ⊙ symbols represent the matrix multiplication and Hadamard (elementwise) product operators, respectively.

As a very simple example of a probabilistic theory of chemistry, we postulated each compound to be capable of having one or more abstract properties to varying degrees, indicated by a number ranging continuously between 0 and 1. The assignment of these abstract properties could be equally interpreted as partitioning the compounds in chemical space into overlapping fuzzy sets (38). A prior distribution is also selected for the reactivity between each set of compounds (Fig. 2*A*). Combining these two distributions gives the joint probability distribution for compounds *α* and *β* to belong to mutually reactive sets *A* and *B* and react as a result (Fig. 2*B*). This formulation can be extended to 3 and 4 component reactions (detailed mathematical formulation in *SI Appendix*).

## In Silico Verification Using Simulated Discovery

**Fig. 3.**
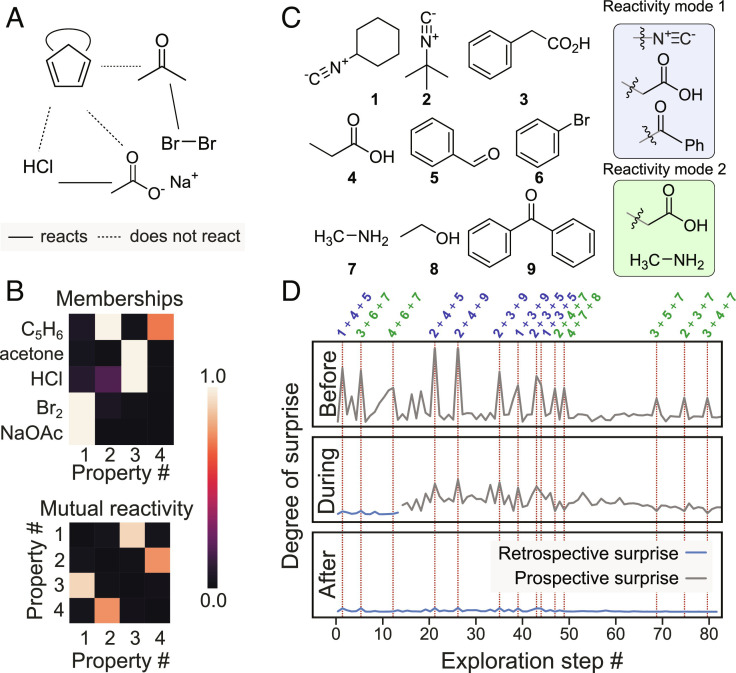
Simulated discovery of the Diels–Alder and Passerini reactions. (*A*) A simple chemical space and associated reactivity observations which could indicate the Diels–Alder reaction. Missing connections indicate combinations whose reactivity has not yet been investigated. (*B*) Compound properties and reactivities inferred by the model by observing the reactivity pattern in *A*. (*C*) Compounds used in the study. Two- and three-component reactions between these compounds made up the chemical space explored. Inferred structural motifs corresponding to reactive fingerprints for the Passerini reaction (reactivity mode 1) and the acid–base reaction between amines and carboxylic acids (reactivity mode 2), (*D*) Evolution of model beliefs throughout the exploration process. The horizontal axis shows the progress of chemical space exploration as consecutive reactions attempted, and the vertical axis represents the degree of surprise (defined as the inverse logarithm of each observation’s likelihood). Observing the Passerini reaction is highly unlikely in prospect at the outset, i.e., a priori (gray trace), but is discovered as a rule by the end of the exploration process, i.e., a posteriori (blue trace).

Before using our system in an experimental setup, we simulated its behavior when given artificial reactivity data. This validation step served to ensure that the output reflected the basic intuition underlying our model. We first looked at the Diels–Alder reaction, notable for motivating the formulation of pericyclic mechanisms. As the Diels–Alder reaction could not be explained using the existing properties known at the time, e.g., acid and base, the labels diene and dienophile had to be devised in order to describe the structural features of its participants. We looked at reactions within a small chemical space of molecules known at the time to test whether the model would be able to make the same deduction (Fig. 3*A*). A typical sample from the model after revealing the reactivity data in Fig. 3*A* is shown in Fig. 3*B*. Cyclopentadiene is seen to possess two mutually reactive properties distinct from those of all other compounds. We were encouraged by this finding since the two properties in question have direct analogues in organic chemistry, i.e., diene and dienophile. In line with the use of a conservative prior distribution for properties, only the minimum (namely four) needed to explain the reactivity observations were used, in accordance with Occam’s razor.

The assignment of molecules to sets does not preclude the use of molecular structures. It is possible to create an alternative probabilistic model that reasons about the chemical structure of molecules by representing each molecule as a vector indicating the presence or absence of certain structural features. These bit string representations, known as molecular fingerprints, are a well-studied subject within the field of cheminformatics (39), and there are several widely used algorithms available (40, 41), notably extended connectivity fingerprints based on the Morgan algorithm (42) and substructure query sets such as MACCS keys (43). Given a suitable bit vector representation, simply adapting the model to assign memberships to each fingerprint bit instead of each molecule enables reasoning about reactivity in terms of structural motifs (see *SI Appendix* for implementation details).

To validate the use of probabilistic reasoning about structural features using structural fingerprints, we constructed an artificial chemical space consisting of 36 two- and 84 three-component combinations between the compounds in Fig. 3*C* (see *SI Appendix* for observations in this dataset). The model was seeded with the outcome of the 36 binary reactions as initial knowledge and tasked with exploring the chemical space by randomly picking one of the remaining experiments at each step. Following this exploration phase, the model was able to infer a three-component reactivity mode, the Morgan fingerprint bits for which correspond to the isocyanide, carboxylic acid, and carbonyl motifs, i.e., the Passerini reaction. Likewise, a binary reactivity mode was inferred related to the carboxylic acid and amine groups (Fig. 3*C*).

By tracking the likelihood of observations—that is, how probable or unsurprising each observation is, whether reactive or nonreactive—as the model explores this chemical space, it is possible to pinpoint when anomalous observations are made and at what point these observations are interpreted as a discovery rather than anomaly (Fig. 3*D*). Once the empirical outcome of an experiment is known, it is possible to compare the likelihood before and after, that is, a priori versus a posteriori. The top graph in Fig. 3*D* shows the model’s degree of surprise to the outcomes before (indicated by the logarithm of observation likelihood) any observations have been revealed to it. As the exploration progresses (middle plot), the model revises its beliefs, accepting the Passerini reaction as predictably reactive rather than anomalous. At the end of the exploration, no observation is indicated as anomalous, meaning the model’s final interpretation is consistent with all outcomes.

## Integration with Robotic Chemistry Platform

**Fig. 4.**
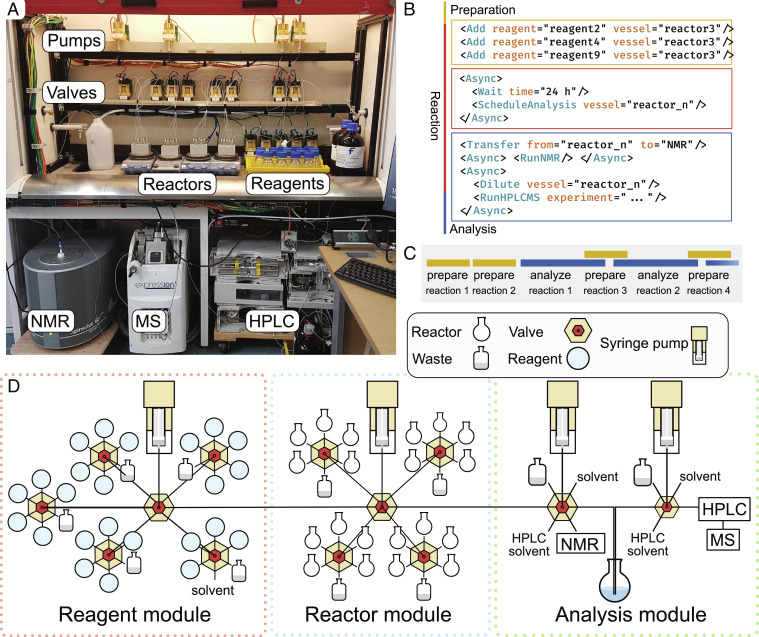
Organization of the robotic chemical platform. (*A*) Physical setup used for discovery. (*B*) Schematic representation of the discovery platform in χDL. (*C*) Experimental workflow for autonomous discovery. Reagents are combined as recommended by the probabilistic algorithm transferred to any available (empty and clean) reactor. Following a set reaction time, the first mixture past the set reaction time is analyzed, and the reactor is cleaned. (*D*) The robotic platform is made of a reagent module holding up to 24 starting materials, a reactor module with 20 flasks and an analysis module. The reagents are mixed into the reactors, heated under inert atmosphere, and analyzed with NMR, MS, and HPLC with diode-array detection (HPLC-DAD).

Probabilistic interpretation is most versatile in combination with a robotic chemistry platform, such as the Chemputer-based setup (Fig. 4*A*). This platform is composed of a set of syringe pumps and valves for liquid handling, dispensing chemicals, moving the reaction mixtures, and cleaning the robot. It is capable of mixing reagents from a pool of up to 24 stock solutions to prepare reactions in 20 concurrent experiments kept under inert atmosphere (Fig. 4*D*).

To control the platform, we also expanded χDL^5^ (a programming language for digital execution of chemistry) to permit a nonlinear sequence of operations, collection of online analytical data such as ^1^H NMR, and real-time decision-making based on the outcome of previous reactions. The current system implements real-time flow benchtop NMR, mass spectrometer, and HPLC. All instruments are remotely controlled using Python libraries that we developed to interface with manufacturer APIs using χDL steps (e.g., “Acquire HPLC”, “Shim NMR”) (Fig. 4*B*). Reaction temperature can also be adjusted using remote-controlled hotplates, allowing each reaction to proceed at constant temperature. χDL can perform the sequence of operations required for reaction setup and analysis in parallel, so the platform is capable of analyzing the contents of one reactor every hour (Fig. 4*C*).

## Experimental Discovery of Historical Chemical Reactions

As a starting point for validating the probabilistic approach to discovery in an experimental setting, we studied the system’s behavior while operating in a chemical space containing a set of historically significant chemical reactions (44–51). The goal was to verify whether the Oracle is able to identify these discoveries and quantify their significance without referring to prior information from the literature. To this end, nine landmark name reactions (listed in Table 1) from a range of periods in the history of synthetic chemistry were considered, and a corresponding set of 11 compounds, which participate in these reactions were selected to use as reagents in our robotic platform (Table 1). We deliberately selected a chemical space with known discoveries so that the oracle’s interpretation could be directly compared and validated against current understanding of these reactions.

**Table 1.** Named reactions contained in the validated chemical space, reactants/reagents involved, and the detected reactivity vectors

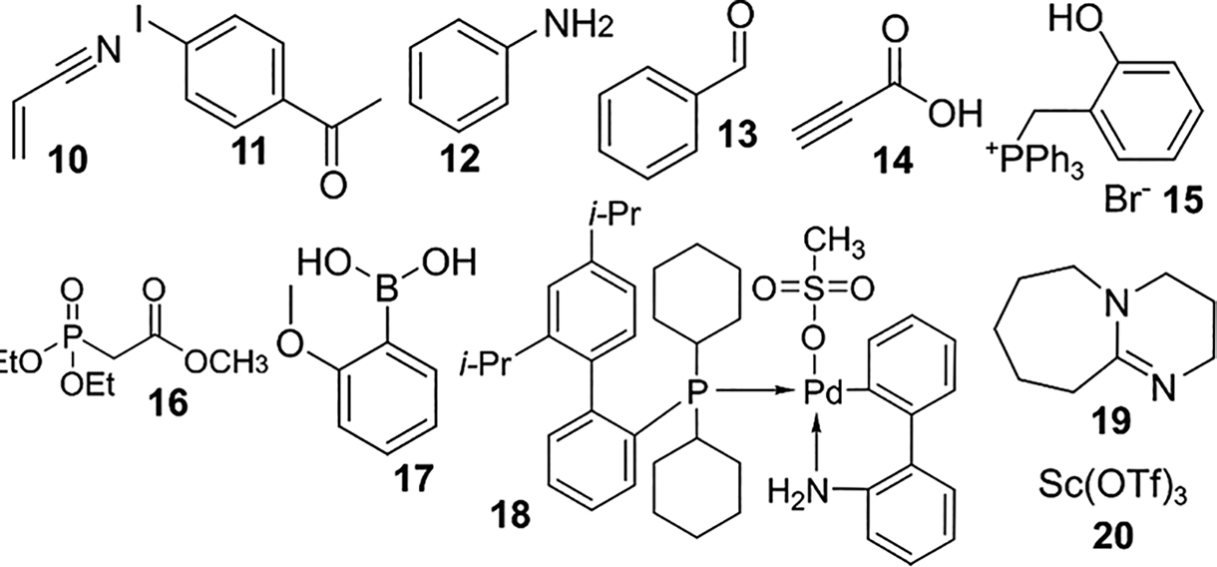					
Reaction	Reactants/reagents				Reactivity vector
Aldol condensation (44)	**11**	**13**	**19**	**–**	0000000**1**
Buchwald–Hartwig amination (45)	**11**	**12**	**18**	**19**	00000**1**00
Heck reaction (46)	**10**	**11**	**18**	**19**	00000**1**00
Mannich reaction (47)	**11**	**12**	**13**	**20**	0100000**1**
Sonogashira reaction (48)	**11**	**14**	**18**	**19**	00001**1**00
Suzuki reaction (49)	**11**	**17**	**18**	**19**	00000**1**10
Wittig reaction (50)	**13**	**16**	**19**	**–**	000001**1**1
Wittig-Horner reaction (51)	**13**	**15**	**19**	**–**	000000**1**0

Digits displayed in bold denote reactivity unique to the reaction in question, i.e., not observed when a subset of reactants/reagents are combined.

A simplification used so far and commonly encountered in systems interfacing robotic platforms with machine-learning algorithms is the use of binary reactivity observations (52, 53)—that is, the outcome of each experiment is simply described as reactive or nonreactive (0 or 1). This restriction prevents reasoning in situations where the precise mode of reactivity is of interest. For instance, with reaction outcomes stored independently as binary labels, it is not possible to know whether A + B + C: 1 signifies a three-component given A + B: 1 also. The Bayesian Oracle is not inherently limited to binary observations, so in the next stage, we devised a multibit vector that allows the description of different types of reactivity. Specifically, we binned the HPLC-DAD retention times of the reactants (prior to the reaction) and final reaction mixture into a set of regions and compared. The presence of new peaks in each region is recorded as a reactivity vector used as the outcome (Table 1 and *SI Appendix*).

## Results and Discussion

We used our system to perform and interpret the outcome of 550 reactions between two, three, or four reactants. At the start of each iteration, the probabilistic model was conditioned on the outcome of all previous reactions, and the most “disruptive” combination—that is, the combination whose outcome would most radically change expected reactivity for the remaining unexplored portion of the chemical space—was selected to perform next. To understand how these historical reactions were discovered and interpreted by the model, we asked whether they were perceived as unexpected when discovered—that is, at the point during exploration when first observed—and whether they seemed justified in retrospect once all reactions had been performed.

**Fig. 5.**
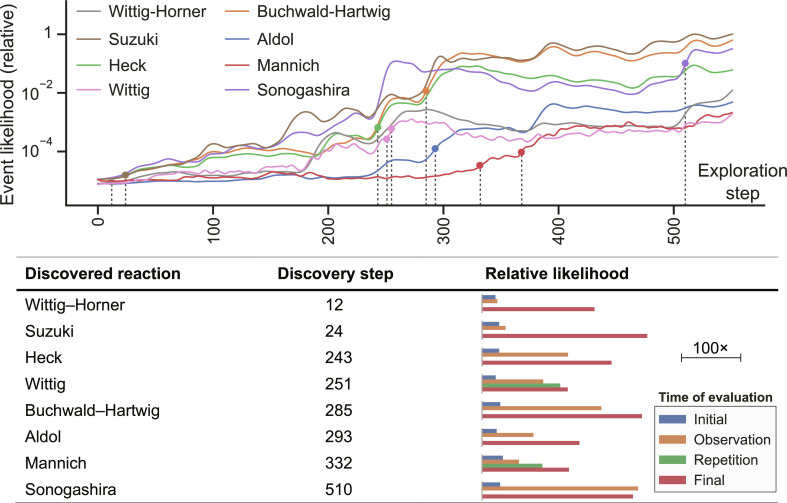
Timeline showing probabilistic interpretation of landmark discoveries. A priori, all reactivities are interpreted as surprising, as shown by their low initial likelihood. During the course of exploration, the model interprets successive reactivity observations and starts to recognize the principal reactivity modes. For instance, the early encounter with the Wittig–Horner reaction is highly surprising, but the Wittig reaction is partially anticipated by the model based on accumulated evidence. Similarly, after discovering the Suzuki reaction, the system anticipates the Heck, Buchwald–Hartwig, and Sonogashira reactions, as evidenced by their high likelihood at the point of observation. The Oracle attempted the Wittig and Mannich reactions twice in order to ensure that they were not anomalies.

Examining the relative likelihood of each discovery (Fig. 5) reveals that the model can make inferences about related reactions. Based on the prior distributions used in this case, all entries are initially seen as highly unlikely. The model quickly learns about the Heck, Buchwald–Hartwig, and Sonogashira coupling reactions following the discovery of the Suzuki reaction at step 24. The Wittig reaction shows a similar dependence on the Wittig–Horner reaction (discovered at step 12); only the first to be discovered is found surprising.

**Fig. 6.**
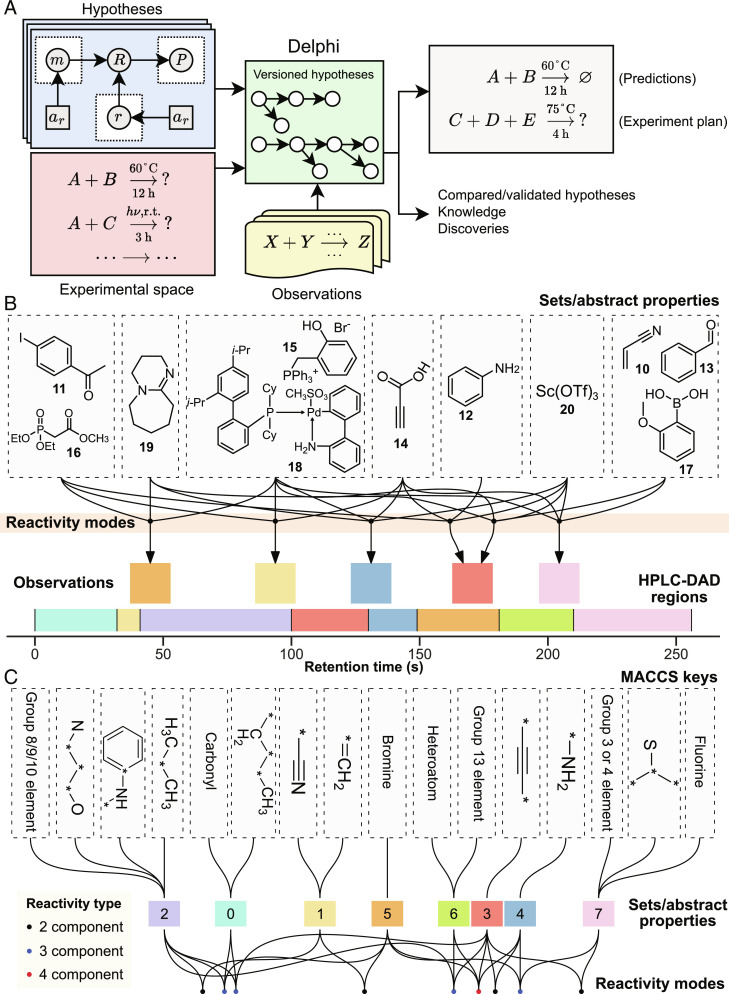
Interpreting the same reactivity observations under different theories using Delphi. (*A*) Delphi’s role in the discovery workflow. (*B*) Visualization of inferred reagent sets and reactivity modes in a structure-free theory. Arrows show the connection between reactivity modes and events, in this case, observation of new HPLC-DAD peaks. (*C*) Visualization of primary structural motifs (as defined by MACCS patterns), the abstract properties conferred by each, and the reactivity modes associated with the interaction of various properties (colors denoting reaction arity). Each reactivity mode is defined by the observation of a unique new peak in the product HPLC-DAD chromatogram.

The theories of chemistry demonstrated so far have been simplified to illustrate the types of insight enabled by automated Bayesian interpretation, but we envision that the greatest utility will be possible within a workflow where theories of much greater detail can be defined and evaluated by domain experts (54). To facilitate and formalize this workflow, we have created *Delphi*, a platform for hosting and interrogating Bayesian theories, Fig. 6*A*. An arbitrary number of hypotheses can be deposited in the Delphi, which assigns them unique identifiers so they can be iteratively refined and derivatized. The same set of results can be interpreted under multiple theories, providing a quantitative and objective means of evaluating the relative merit of rival theories (55–57). As a demonstration, we reinterpreted the robot’s experimental findings under an alternative theory that links structural motifs in participating reagent molecules (represented by their MACCS keys) to the number of unique product HPLC peaks in each reaction (rather than their location). The resulting interpretations are compared side by side in Fig. 6 *B* and *C*.

Founded upon recent progress in laboratory automation and online analytics, the long march toward digitizing chemical discovery has passed a number of landmark developments involving varying degrees of reliance upon and interpretability by human chemists. With this work, we demonstrate that eliminating expert input is not a necessary condition for removing hidden bias or using modern hardware to reason about reactivity in large chemical spaces. Present probabilistic methods still present computational challenges when exploring hypothesis spaces parameterized by a large number of latent dimensions or discrete parameters. Defining bespoke probabilistic models to represent domain knowledge also requires familiarity with probability theory and the inference method used. We expect the steady improvement of both inference algorithms and probabilistic programming languages to lower the computational as well as cognitive barriers to wider adoption of the probabilistic paradigm. Meanwhile, progress toward a shared standard for describing the experimental space (inputs) and predictions (outputs) will allow systems like Delphi to act as repositories for reusable expert chemical knowledge, facilitating reproducible collaboration on discovery campaigns carried out around the globe.

## Methods

### Probabilistic Modeling.

Inference is carried out using Hamiltonian Monte Carlo, specifically using the No-U-turn sampler (34) algorithm for sampling as implemented in the NumPyro probabilistic programming package (33, 58), early prototyping performed using the PyMC3 probabilistic programming package (59). The methodology for Bayesian model comparison was based on the work of Kamary *et al*. through a combination of candidate models into a mixture model and inspection of the posterior mixing distribution (57). *SI Appendix* contains plate diagrams as well as prior and likelihood distributions for all models used.

### Robotic Platform.

Full specification regarding individual devices and their organization within the robotic platform as well as vendor information for online analytics is provided in *SI Appendix*.

## Supplementary Material

Appendix 01 (PDF)

## Data Availability

*SI Appendix* contains Materials and Methods, mathematical formulation of probabilistic model, software implementation of probabilistic Oracle and Delphi, and software implementation of the robotic platform. Our implementation of the Chemical Oracle is available online at https://github.com/croningp/chem_oracle. A dataset containing the experimental reactivity data in the explored chemical space is freely available on Zenodo: https://zenodo.org/record/6337271.
