# Supplementary material for: Digitizing chemical discovery with a Bayesian explorer for interpreting reactivity data
Source: Proc Natl Acad Sci U S A. 2023 Apr 17;120(17):e2220045120. doi: 10.1073/pnas.2220045120 (PMC10151610; doi:10.1073/pnas.2220045120)
Supplement: Supplementary file 1 — Appendix 01 (PDF) [file pnas.2220045120.sapp.pdf]

Supplementary Material for:

Digitizing Chemical Discovery with a Bayesian Explorer for Interpreting Reactivity Data

S. Hessam M. Mehr†, Dario Caramelli†, Leroy Cronin*

School of Chemistry, University of Glasgow, Glasgow, G12 8QQ, UK.

*Correspondence to: Lee.Cronin@Glasgow.ac.uk

Contents

1. Bayesian model 3

1.1. Overview 3

1.2. Model description 3

1.3. Priors 5

1.3.1. Non-structural model with stick-breaking membership prior 6

1.3.2. Structural model 6

1.4. Deterministic and derived quantities 7

1.5. Inference 8

2. Physical setup 9

2.1. Liquid handling robot 9

2.2. Reagents module 12

2.3. Reactors module 12

2.4. Analysis module 13

2.4.1. HPLC 13

2.4.2. MS 15

2.4.3. NMR 16

3. Platform execution 18

3.1. Interfacing with analytical instruments 18

4. Reactivity detection 18

4.1. HPLC reactivity 18

5. Experimental validation 21

6. Results inferred from probabilistic model 23

7. References 26

1. Bayesian model

1.1. Overview

The central concept is a set of N abstract properties. Each compound is therefore described by an N-dimensional *membership vector* $\boldsymbol{m_A}$, whose entries $0 < m_{Ai} < 1$ describe to what degree compound A possesses abstract property i or, alternatively, to what degree compound A belongs to the fuzzy set i. We use a conservative prior known as the *Dirichlet process*, specifically it's *stick-breaking construction,* in order to allocate properties to each compound. The conservative nature of this prior means the expectation value (EV) $E(m_{Ai})$ is a decreasing function of i.[1] As a result, only a finite number of properties will get be non-negligibly allocated for sufficiently large N, meaning of all practical purposes N can be assumed to be infinite. The choice of the stick-breaking process is in accordance with Occam's razor, creating new sets only when the current allocation cannot adequately explain observations.

Corresponding to the concept of memberships is the mutual reactivity of the various properties. This is described by the reactivity matrix $\boldsymbol{R}$ where $R_{ij} = R_{ji}$ and $R_{ii} = 0$, *i.e.* reactivity between properties is symmetric and properties are not reactive towards themselves. The element-wise (also known as Hadamard) product of $\boldsymbol{R}$ with the outer product of memberships vectors for compounds A and B, $\boldsymbol{M_{AB}} = \boldsymbol{m_A} \otimes \boldsymbol{m_B}$ results in matrix $\boldsymbol{X_{AB}} = \boldsymbol{R} \odot \boldsymbol{M_{AB}}$. The entries $\boldsymbol{X}_{\boldsymbol{AB},ij}$ describe the probability of reaction between A and B due to A's property i and B's property j. The overall probability of reaction between A and B can then be calculated as $P_{AB} = 1 - \prod_{ij}(1 - \boldsymbol{X}_{\boldsymbol{AB},ij})$, that is, the complement of the probability that no combination of properties leads to a reaction. Reactions between three or four compounds can similarly be described by extending this formulation to rank 3 or 4 tensors (see SI).

1.2. Model description

A total of four different probabilistic models were used. A high-level graphical model description of the models used in this work is shown as plate diagrams in **Figure 1**–**Figure 4**, using an adaptation

of the literature notation.[2,3] The quantity P is defined in Section 1.4. Symbols enclosed in circles represent random variables while hyperparameters are shown within squares. Grey shading denotes observed quantities.

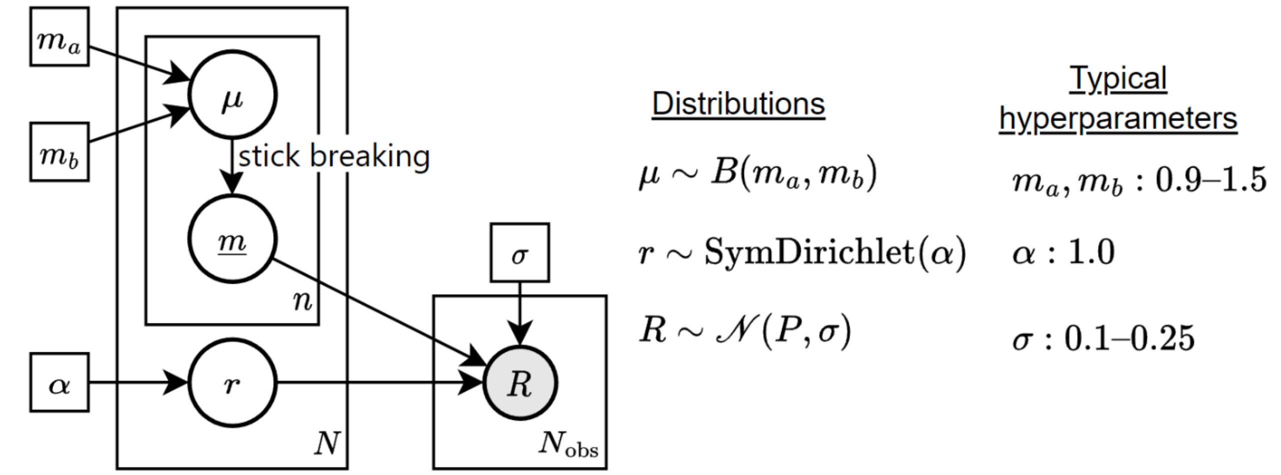

Figure 1. Graphical model for non-structural model with Dirichlet reactivity prior.

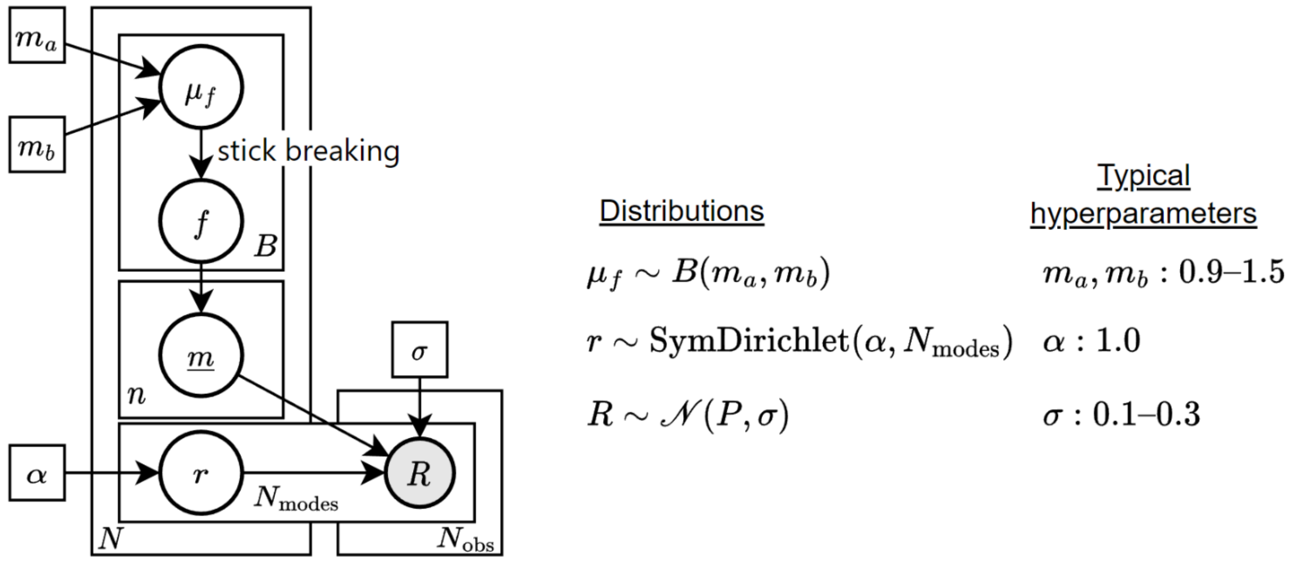

Figure 2. Graphical model for structural model with Dirichlet reactivity prior.

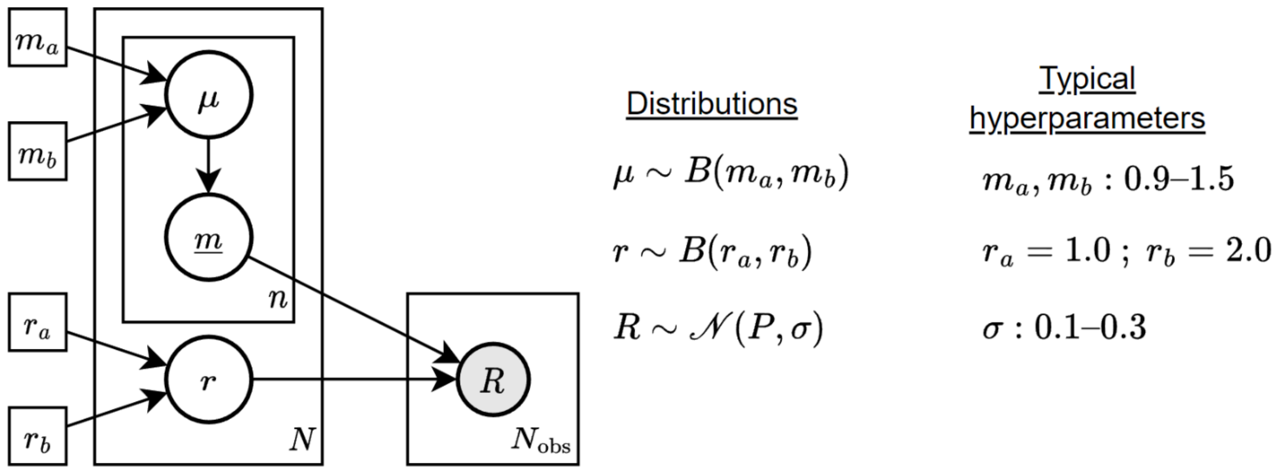

Figure 3. Graphical model for non-structural model with simple beta reactivity prior.

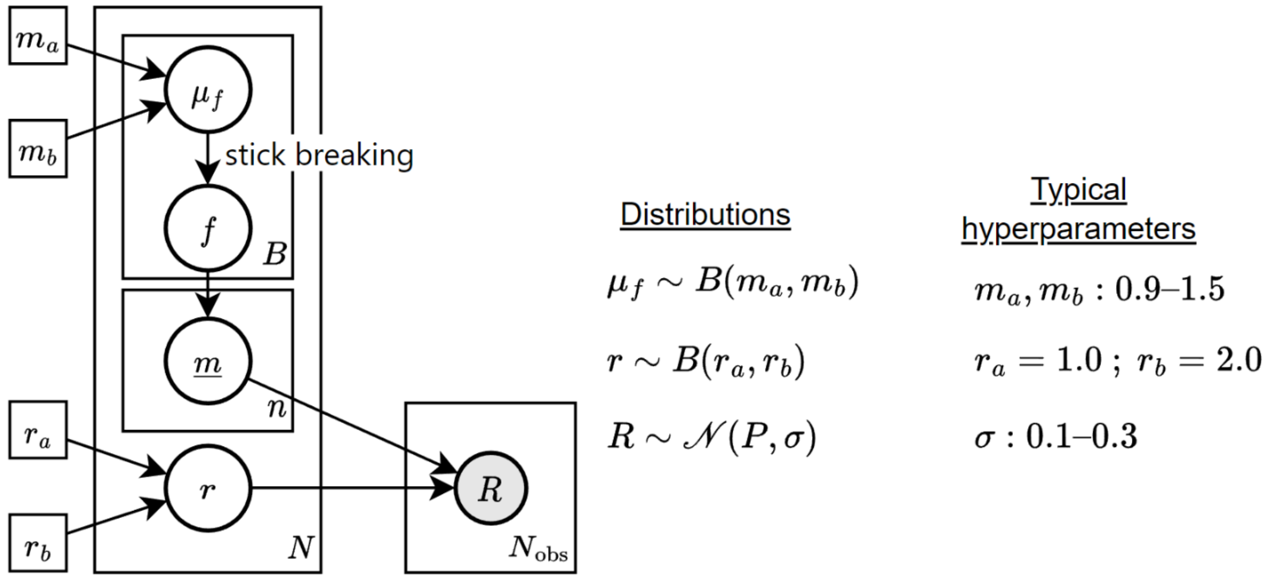

Figure 4. Graphical model for structural model with simple beta reactivity prior.

1.3. **Priors**

Each compound X is assumed to possess a number of properties, m_{Xi}. In the non-structural model, these properties are assigned (sampled) directly without reference to the chemical structure of the compound. The structural model instead assigns properties f_{Xi} to each fingerprint X. The properties of the compound are then derived deterministically by combining f_{Xi} with the fingerprints present in the molecule.

1.3.1. Non-structural model with stick-breaking membership prior

Set memberships are sampled, using the stick-breaking construction, from a Dirichlet process with concentration factor α = 5.0 and a beta base distribution with parameters α = 1.0 and β = 5.0.

To account for compounds that have no properties, an extra "dummy" property (corresponding to a property with no reactivity) is sampled at this point, the membership for which is disregarded during reactivity calculations. Before discarding this property, the membership vector for each compound is normalized so the largest membership is equal to 1.0, *i.e.* normalized according to L_∞. This normalization step allows the same compound to belong to more than one reactive set with high probability if necessary, reflecting the intuitive notion that some compounds may possess more than one property.

In the case of simple binary observations (reaction or no reaction), the unique members of the symmetric reactivity tensor, *e.g.* R_{ij} $(i > j)$ come from a beta distribution between 0 and 1, $\mathcal{U}_{[0,1]}$. For 3- and 4-component reactions, a similar rank-3 or rank-4 distribution is used ($R_{i>j>k} \sim \text{Beta}(\alpha, \beta)$ and $R_{i>j>k>l} \sim \text{Beta}(\alpha, \beta)$).

The later examples in the manuscript, where reactivity is described by a vector of events corresponding to different types of reactivity, required the addition of an extra dimension to each reactivity matrix, *i.e.* $R_{ijx}, R_{ijkx}, R_{ijklx}$. The *x* axis is the same dimension as the number of possible reactivity observations, *i.e.* the length of the reactivity vector. The prior distribution is independent and identically distributed (i.i.d.) for different *i*, *j*, and *k* but Dirichlet distributed with respect to the *x* axis, *i.e.* $\sum_x R_{ijx} = R_{ijkx} = R_{ijklx}$. This choice of prior reflects the belief that properties generally contribute to a limited number of reactivity observations.

1.3.2. Structural model

Fingerprint memberships f_{xj} are sampled from a beta distribution with $\alpha = 1$ and $\beta = 3$. The probability distribution function (PDF) of this distribution is shown below and corresponds to the belief that most structural motifs have a small contribution to the properties of a given compound.

1.4. Deterministic and derived quantities

In the structural model, where compound membership m_{Xi} are not sampled directly, they are calculated as follows for a given compound *X*

$$m_{Xi} = \max_{j} F_{Xj} f_{ji}$$

$$= \max\{f_{ji} \mid F_{Xj} = 1\}$$

Here F_{Xj} refers to the *j*th bit in the molecular fingerprint of compound *X*. In this work, we use 256-bit Morgan fingerprints with a radius of 1. This equation expresses the model where the degree of membership is the maximum conferred by any of the structural motifs present in the molecule.

For a given reaction between compounds *X* and *Y*, the probability that they react is calculated as the complement of the probability that no pair of properties leads to a reaction.

$$P_{XY} = 1 - \left[\prod_{ij} \left(1 - m_{Xi} r_{ij}\right)\right]$$

The probability of reaction between three chemicals (*X*, *Y*, and *Z*), and four chemicals can be calculated in a similar fashion.

$$P_{XYZ} = 1 - \overline{P}_{XY}\overline{P}_{XZ}\overline{P}_{YZ}\overline{P}_{XYZ}$$

$$P_{XYZT} = 1 - \overline{P}_{XY}\overline{P}_{XZ}\overline{P}_{XT}\overline{P}_{YZ}\overline{P}_{YT}\overline{P}_{XYZ}\overline{P}_{XYT}\overline{P}_{XZT}\overline{P}_{YZT}\overline{P}_{XYZT}$$

Where $\overline{P}$ signifies complement probability, *e.g.* $\overline{P}_{XY}$ the probability that no binary reaction happens between *X* and *Y*.

$$\overline{P}_{XY} = \prod_{ij} \left(1 - m_{Xi} m_{Yj} r_{ij}\right)$$

$$\overline{P}_{YZ} = \prod_{ij} \left(1 - m_{Xi} m_{Yj} r_{ij}\right)$$

$$\overline{P}_{XZ} = \prod_{ij} \left(1 - m_{Xi} m_{Yj} r_{ij}\right)$$

$$\overline{P}_{XYZ} = \prod_{ijk} \left(1 - m_{Xi} m_{Yj} m_{Zk} r_{ijk}\right)$$

$$\overline{P}_{XYZ} = \prod_{ijk} \left(1 - m_{Xi} m_{Yj} m_{Zk} r_{ijk}\right)$$

$$\overline{P}_{XYT} = \prod_{ijk} \left(1 - m_{Xi} m_{Yj} m_{Tk} r_{ijk}\right)$$

$$\overline{P}_{XZT} = \prod_{ijk} \left(1 - m_{Xi} m_{Zj} m_{Tk} r_{ijk}\right)$$

$$\overline{P}_{YZT} = \prod_{ijk} \left(1 - m_{Yi} m_{Zj} m_{Tk} r_{ijk}\right)$$

$$\overline{P}_{XYZT} = \prod_{ijkl} \left(1 - m_{Xi} m_{Yj} m_{Zk} m_{Tl} r_{ijkl}\right)$$

1.5. **Inference**

Inference was carried out using Hamiltonian Monte Carlo (HMC), specifically using the No-U-turn sampler (NUTS) algorithm for sampling as implemented in the *numpyro* probabilistic programming package,[4,5] early prototyping performed using the PyMC probabilistic programming package.[6] The following parameters were used in conjunction with NUTS sampling.

$$\text{Acceptance probability} = 0.65$$

$$\text{Maximum binary tree depth} = 8$$

2. Physical setup

2.1. Liquid handling robot

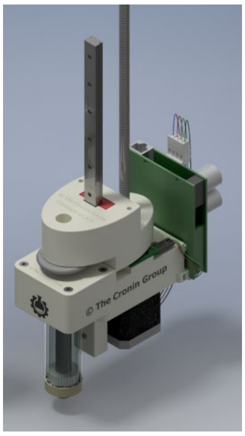

4 x Chemputer Pumps, 5ml syringes

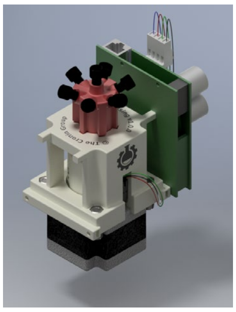

13 x Chemputer Valves, equipped with 1/16" valve head.

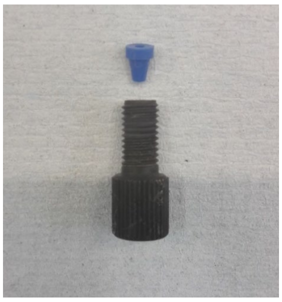

Flangeless Fitting, for 1/16" OD Tubing, 1/4"-28 Flat Bottom, PEEK/ETFE (IDEX-HS XP-218BLK)

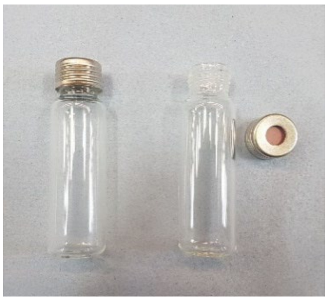

20 x 20 ml clear glass vial. Part No. SU860097, with septum screw caps Part No. SU860102 (Sigma-Aldrich).

Stirrer bars, PTFE coated, 13x3 mm (Fisher)

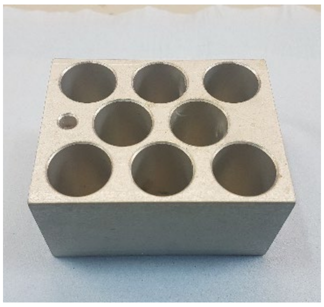

4 x heating blocks, 8 holes, 23.8mm diameter. Part No. 460-3284 (VWR)

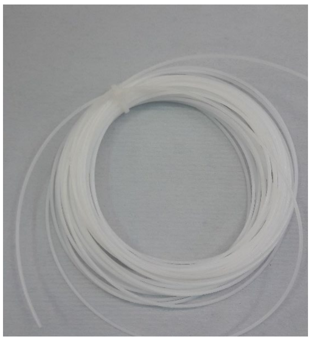

Tubing, PTFE, 1/16" (1.6mm) OD x 0.8mm ID, 20m Part No. 008T16-080-20 (Kinesis)

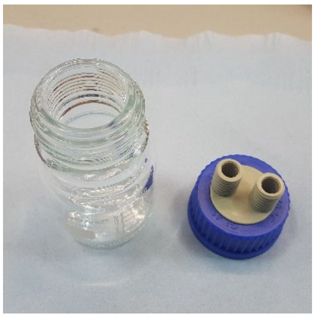

24 x Duran® laboratory bottles, 100 mL, Part no. Z305170-10EA (Sigma-Aldrich). GL 14 screw cap Part No. 215-2082 (VWR), with insert Part No. 215-2077 (VWR)

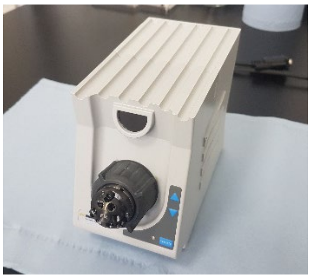

Remote controlled switch valve, two positions. Part no. MXP7920-000 (Rheodyne)

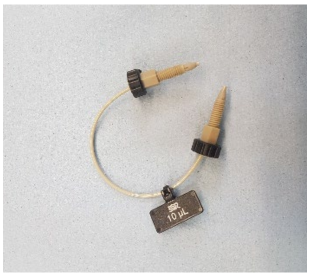

10 µl sample loop, part no. HYPE9055-021 (VWR)

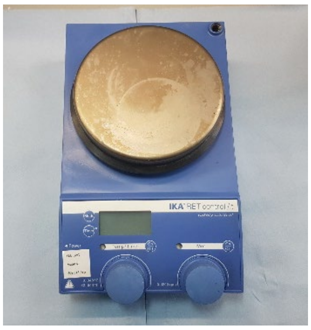

4 x Heating plates. Remote controlled. Part no. either 0005020002 or 0003810002 (IKA)

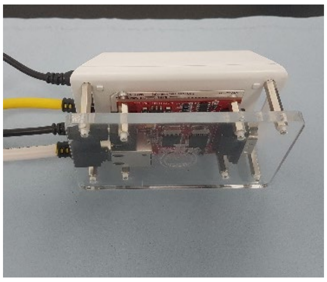

Serial-to-Ethernet Converter, Chemputer part.

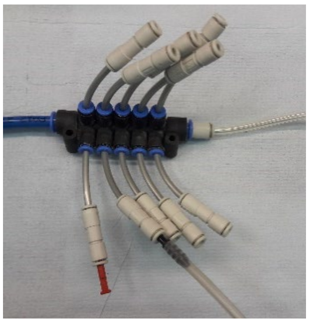

Pneumatic manifold with ten outlets (SMC Corporation, Japan), fitted with 10 non-return valves (SMC Corporation, Japan) and connected to the line through black pneumatic tube-to-tube adapters (Legris, UK) or shut with red blanking plugs (Legris, UK)

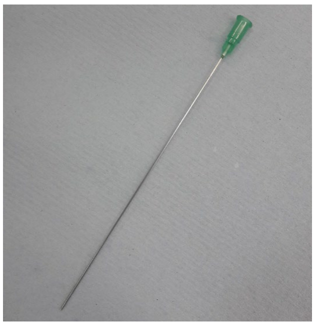

Needles, Sterican, length 120mm

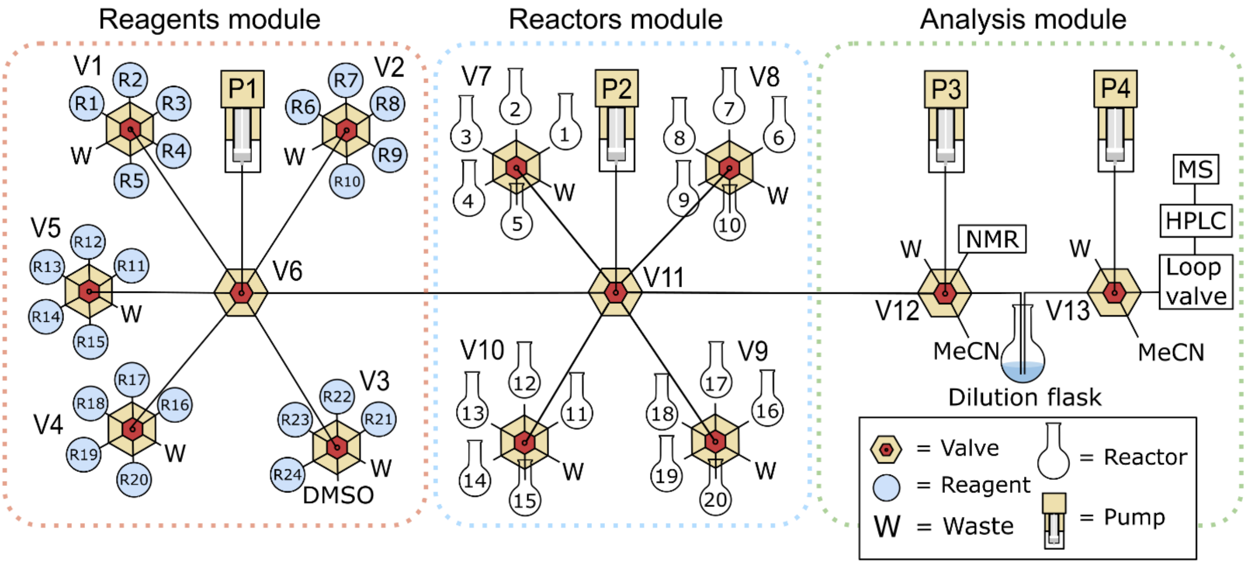

Figure S5: Scheme of the platform configuration highlighting the Reagents module, the Reactors module and the Analysis module.

2.2. Reagents module

This module allows the management of up to 25 reagents bottles. It is made of a pump and five valves (V1-2-3-4-5) arranged in a “star” configuration around a central valve (V6). V1-2-3-4-5 are connected to four reagents each (R1-21) and to V6 through the top port. V6 is connected to the backbone (V11) and pump P1. The fifth port of valves V1-2-3-4-5 is connected to waste and this connection is used to clean the syringe, the valve heads and the connections by pumping a clean solution through it. The platform effectively uses 24 reagents since one of the ports is used for the cleaning solution (DMSO) bottle.

2.3. Reactors module

This module has the same configuration of the Reagents module and manages up to 20 reactors. V11 is connected to the backbone (V12 and V6) and pump P2. The other four ports are connected to valves V7-8-9-10. At the beginning of each reaction reagents are pumped into the reactors in sequence from the Reagents module. The lines, pumps and valves are then cleaned by pumping cleaning solution to the waste port connected to the valve that is managing the reactor. The reactors are equipped with

magnetic stirring bars and placed inside a heating block on top of a stirring plate kept at 30 ℃. During the last part of the project the reactors are heated at 60 ℃ and kept in inert atmosphere by continuously bubbling nitrogen through the solutions. After the reaction is completed the mixture is pumped back to P2 and moved through the backbone to the analysis module.

2.4. **Analysis module**

This module is made by two valves, two pumps, a dilution flask, the switch valve and the analysis instruments (HPLC-MS and NMR). Valve V12 is connected to the backbone (V11), the NMR flow cell, the dilution flask and a solvent bottle (MeCN). V13 and P4 are connected to the dilution flask and the switch valve and are purposely kept isolated from the backbone to maintain the components as clean as possible since they manage the HPLC injection. During the analysis 4 ml of the reaction mixture are pumped from the reactors module into the syringe of pump P3. 0.3 ml of solution are pumped into the dilution flask while the remaining is pumped into the NMR flow-cell. The solution is diluted with MeCN and pumped using V13 and P4 into a remotely controlled switch valve equipped with a 10 µl sample loop. Once the valve is switched, the HPLC is triggered and the method is run. Between each experiment the procedure was repeated injecting clean MeCN in order to collect a blank. Pumps and valves are cleaned by flushing them with MeCN. The NMR flow-cell is cleaned by flushing it with DMSO.

2.4.1. HPLC

In order to move exactly 0.3 ml of reaction mixture the dilution flask is connected with two lines to valve V12: line A and B. Line A was purposely cut to have exactly 0.3 ml of dead volume. During the dilution procedure an excess of mixture (0.6 ml) is pumped to the dilution flask through line A. The flask is then emptied from line B and 6.5 ml of acetonitrile are pumped into it through line A, pushing the sample from the tubing into the flask. The mixture is then stirred for 30 seconds and pumped into the switch valve with pump P4 (**Figure S6**).

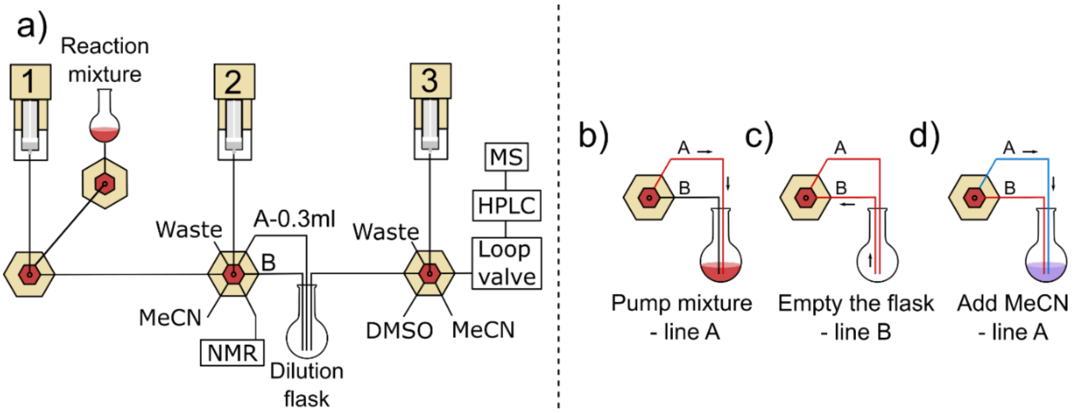

Figure S6: a) scheme of the part of the Chemputer responsible of sampling the reaction mixture, diluting and injecting into the HPLC-MS. b) Procedure to sample exactly 0.3 ml of solution.

When the loop valve is remotely switched 10 µL of the sample are injected in the instrument. The method runs a linear gradient mixture of solvents: water w/0.1% v/v formic acid and acetonitrile w/0.1% v/v formic acid at 0.5 mL per minute, over 21 minutes as indicated in **Table S1**. The column compartment is maintained at 30 °C. UV detection is performed using a diode array detector (DAD) set on 254nm. The output line from the HPLC is split and a fraction is continuously injected into the MS instrument.

Table S1: HPLC method used for the analysis of the reaction mixture.

Time [min]	Water [%]	Acetonitrile [%]
**0**	95	5
**18**	5	95
**21**	5	95
**25**	95	5

2.4.2. MS

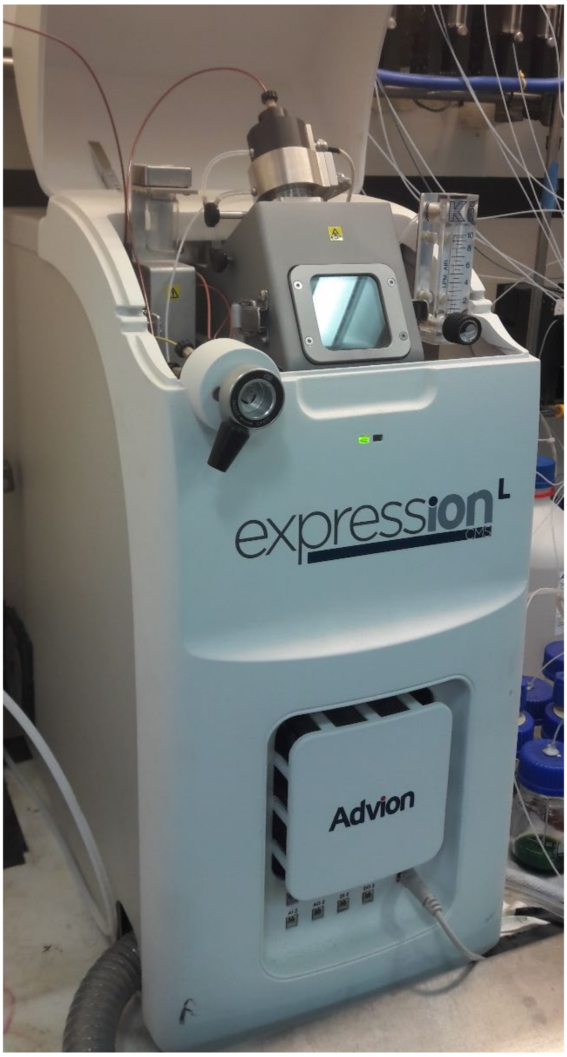

Figure S7: Advion expression-CMS mass spectrometer used in this work.

The Advion expression CMS mass spectrometer system, Figure S7, used in this work has the following specifications.

• Dimensions: 66 x 28 x 56 cm

• Weight: 32 kg

• Gas requirements: Nitrogen 98% pure, 4.1 Bar, 8L/min

• Flow rate range: 10 µL/min to 2 mL/min

• Polarity: Positive & Negative ion switching in single analysis

• m/z Range: 10 to 2,000 m/z

• Resolution: 0.5 - 0.7 m/z units (FWHM) at 1000 m/z units sec-1 over entire acquisition range

• Accuracy: ± 0.1 m/z units over entire acquisition range

• Linear dynamic range of 5 x 10^3

ESI parameters

• Capillary temperature(V) 250.0

• Capillary Voltage(V) 180.0

• ESI Gas Temperature: 250℃

• ESI Voltage: 3500V

• Calibration: Agilent ESI Tuning mix G2421A

The mass spectrometer was connected in series after the HPLC. The HPLC output line was connected to a T splitter to reduce the flow rate and directly injected into the MS. The instrument was calibrated monthly. The synchronisation of the acquisition with the HPLC method run was managed by the main script that orchestrated the platform. In other words, the script would just start the acquisition on both instruments at the same time.

2.4.3. NMR

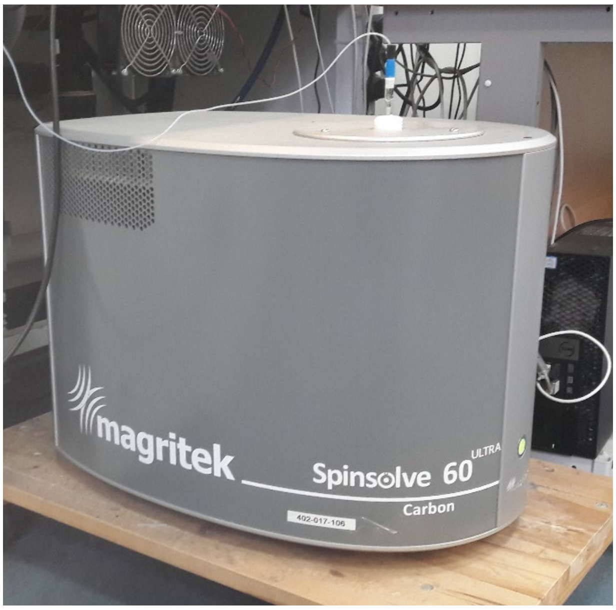

Figure S8: Magritek SpinSolve 60 Ultra spectrometer used in this work.

The Magritek SpinSolve 60 Ultra spectrometer system, Figure S8, used in this work has the following specifications.

• Frequency: 60 MHz Proton

• Resolution: 50% linewidth < 0.5 Hz

• Lineshape: 0.55% linewidth < 20 Hz

• ^{1}H Sensitivity: >120:1 for 1% Ethyl Benzene

• Dimensions: 58 x 43 x 40 cm

• Weight: 60 kg

• Magnet: Permanent and cryogen free

• Stray field: < 2 G all around system

The instrument is equipped with a flow-cell to allow online analysis. The cell goes through the instrument and its location places the NMR tube part at the centre of the magnets. Both inlet and outlet are connected to normal PTFE tubing with screw caps. The flow cell allows automatic reaction monitoring in real time by pumping at least 3 ml of solution from the reaction mixture. The other side of the flow cell is connected to waste, this allow fast and efficient cleaning of the cell by solvent continuous flushing.

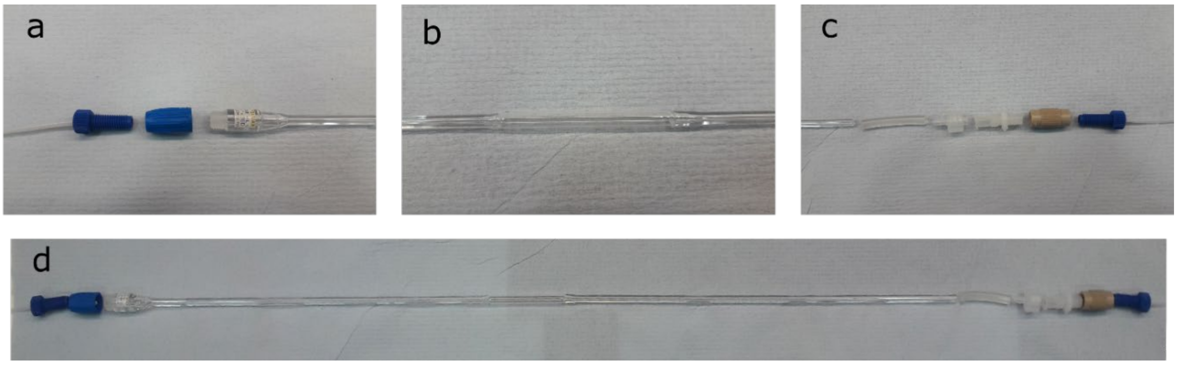

Figure S9: a) top connector, the screw connector has been welded to the glass cell by the glassblower. b) Central part, it has been made by welding a normal 5 mm NMR tube to the rest of the cell. c) bottom connection, the glass could not have the threading because the whole cell needs to fit through the instrument. The connection is made with a chemical resistant tubing followed to a plug to syringe, a syringe to male connector, female to female and finally a normal screwed connector. d) entire NMR flow cell.

3. Platform execution

3.1. Interfacing with analytical instruments

For communicating with the Advion Expression CMS mass spectrometer, we created Python wrappers around the binary libraries supplied by the vendor. This binding is part of our AnalyticalLabware Python library and can be used for controlling the instrument as well as reading the data files produced as a result of MS experiments.

AnalyticalLabware was also used to communicated with the SpinSolve benchtop NMR used in our system. In this case, we use a TCP connection to communicate with the API exposed by the vendor-provided desktop software.

The Rheodyne sampling valve was controlled with Python through serial connection.

The HPLC was controlled using a MACRO that iteratively reads a text file and runs a function (ex: run method, turn on, turn off) related to a key word. The file is automatically updated from Python during the platform loops.

4. Reactivity detection

4.1. HPLC reactivity

In order to reliably recognise the peaks of the starting materials in the chromatogram of the reaction mixture their shift was optimised. The chromatogram regions corresponding to the reagent peaks were isolated and a shift value was assigned to them. By combining the peaks of the individual reagents the software was able to reconstruct a virtual spectrum corresponding to the sum of the starting material and the precise position of each peak was adjustable with the shift values. The real chromatogram was then compared with the reconstructed one and the root mean square (RMS) between the two was calculated. This process was repeated iteratively by an optimization algorithm (the default implementation in SciPy[7]) trying different shifts until the values that lead to the best chromatogram reconstruction were found. During the optimization a loss function was calculated based on the RMS and the values of the shifts themselves. In order to keep the values within

reasonable retention time shifts a threshold of 1 minute was applied meaning that a shift beyond this value would inevitably correspond to a high loss value. The reconstruction worked extremely well in case of no reactivity as the reaction data was indeed a combination of the starting materials while in case of a reaction the best approximation was provided. The peak in the chromatograms were found with the peak finding functionality in the SciPy package using a threshold of 200mAU.

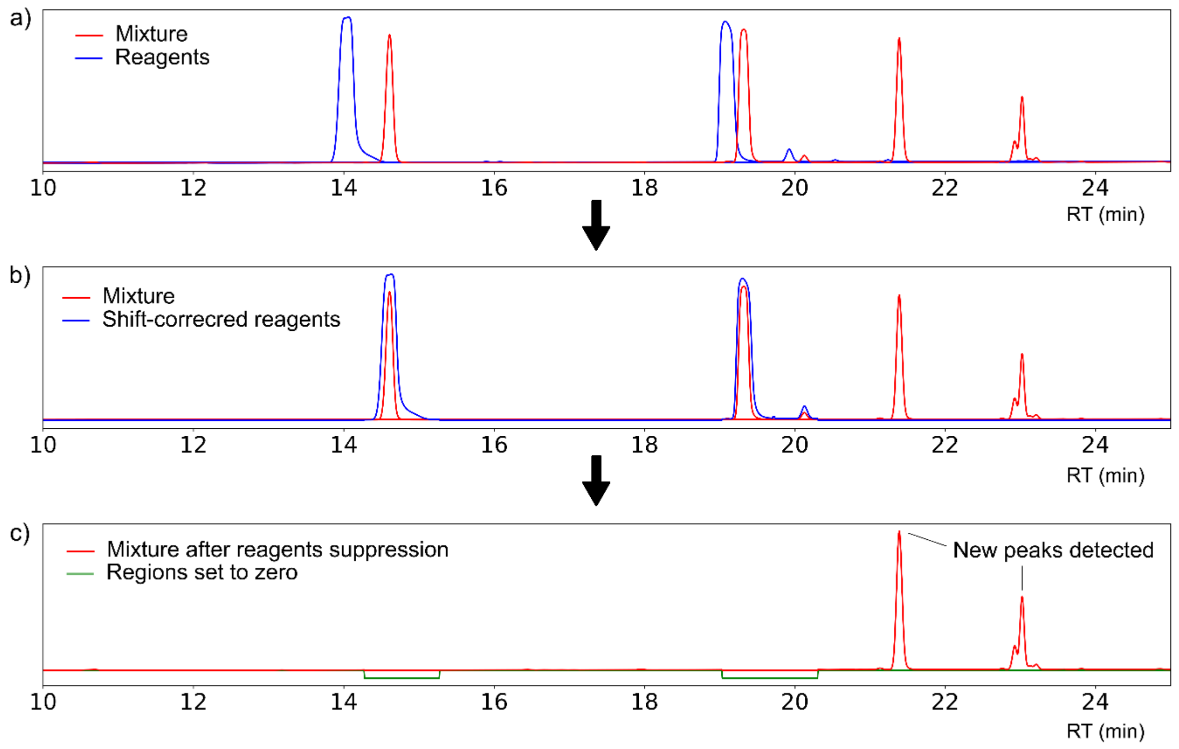

Figure S10: Example of HPLC data processed for reactivity classifications, assigned as reactive mixture. a) the mixture chromatogram and the reagents superimposition. b) The reagents peaks are shifted to optimize the overlap with the mixture. c) The regions corresponding to the reagents peaks are set to zero. Remaining peaks are detected for reactivity.

The data acquired in chemical spaces was processed to find the retention times of all the peaks in the various mixtures. These values were used to generate the position of eight bins that would be used to calculate the reactivity vectors. The bins are positioned in a way where, considering the entire peaks dataset, each bin would on average contain the same number of peaks, using the cumulative distribution of all peaks as reference (Figure S11).

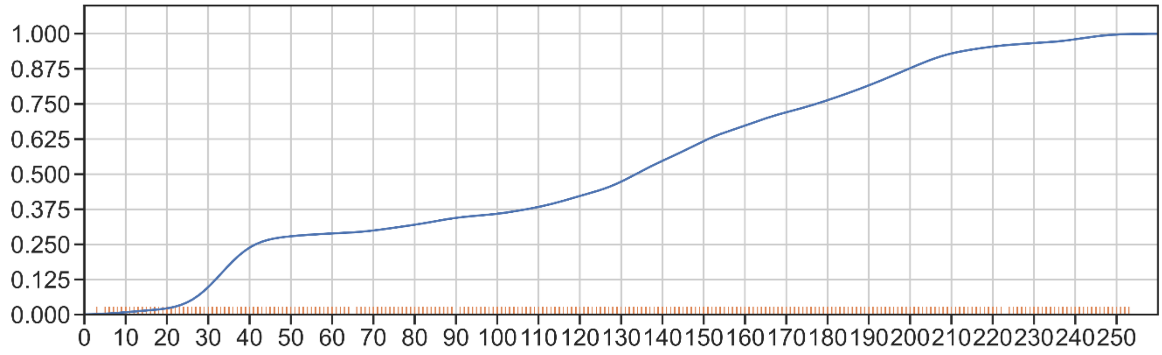

Figure S11. Cumulative distribution of HPLC peak positions over all three chemical spaces explored. This distribution was used in the context of splitting the time axis into equally populated regions.

Examining the reactivity vector for Buchwald–Hartwig amination (**11** + **12** + **18** + **19** in Figure S12a) illustrates how specific reactivity types can be captured in the presence of other reactions,. Even though many sub-combinations of the same reactants were also reactive — that is the product HPLC-DAD spectrograms contain regions with new peaks, the Buchwald–Hartwig reaction can still be detected as a unique reactivity mode based on activation of the sixth entry in its reactivity vector.

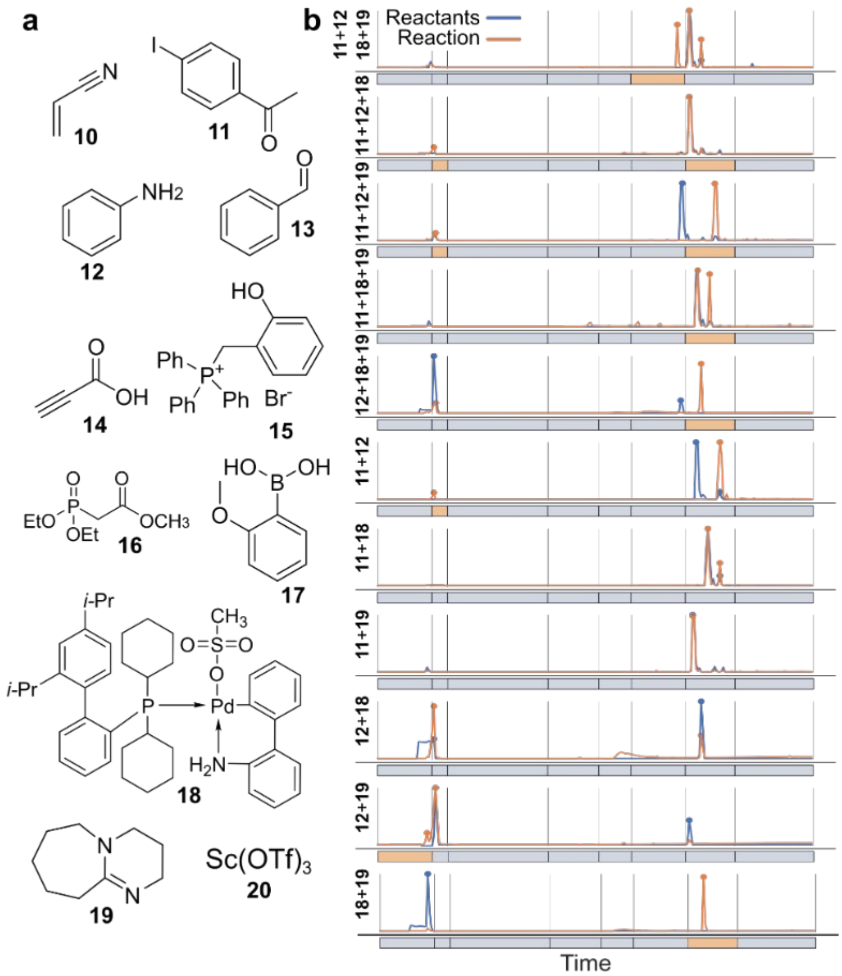

Figure S12. a, Reagents used for experimental discovery. **b**, HPLC–DAD reactivity vectors for the various combinations of **11**, **12**, **18**, and **19**, including the Buchwald–Hartwig reaction (**11** + **12** + **18** + **19**). Each plot depicts the HPLC–DAD spectrogram of the reaction mixture prior to and following the reaction. Spectrogram regions containing new peaks are marked reactive and recorded as a 1 in the reactivity vector (indicated by orange shade underneath).

5. Experimental validation

The rediscovery space was designed to contain the highest number of famous named reactions in the least number of reagents. 11 molecules were selected that would yield 8 known reactions. Most of the reagents were stored in 1M stock solutions in DMSO. DBU was stored as 2M, Sc(OTf)3 as 0.1M and XPhos Pd G3 as 0.05M. Reagents were mixed in equal amount into the reactors keeping the total volume at 4 ml (2ml for a 2 components reaction, 1.3 for 3 components and 1ml for 4 components). The reactors were constantly kept at 50℃ and under inert atmosphere by slow bubbling of nitrogen.

After 19 hours they were automatically analysed with online NMR, MS and HPLC using the method already described.

Before the platform run the reactions were manually tested and analysed with an offline HPLC-MS system. All expected products were confirmed to be present in the reaction mixture as reported in Figure S13.

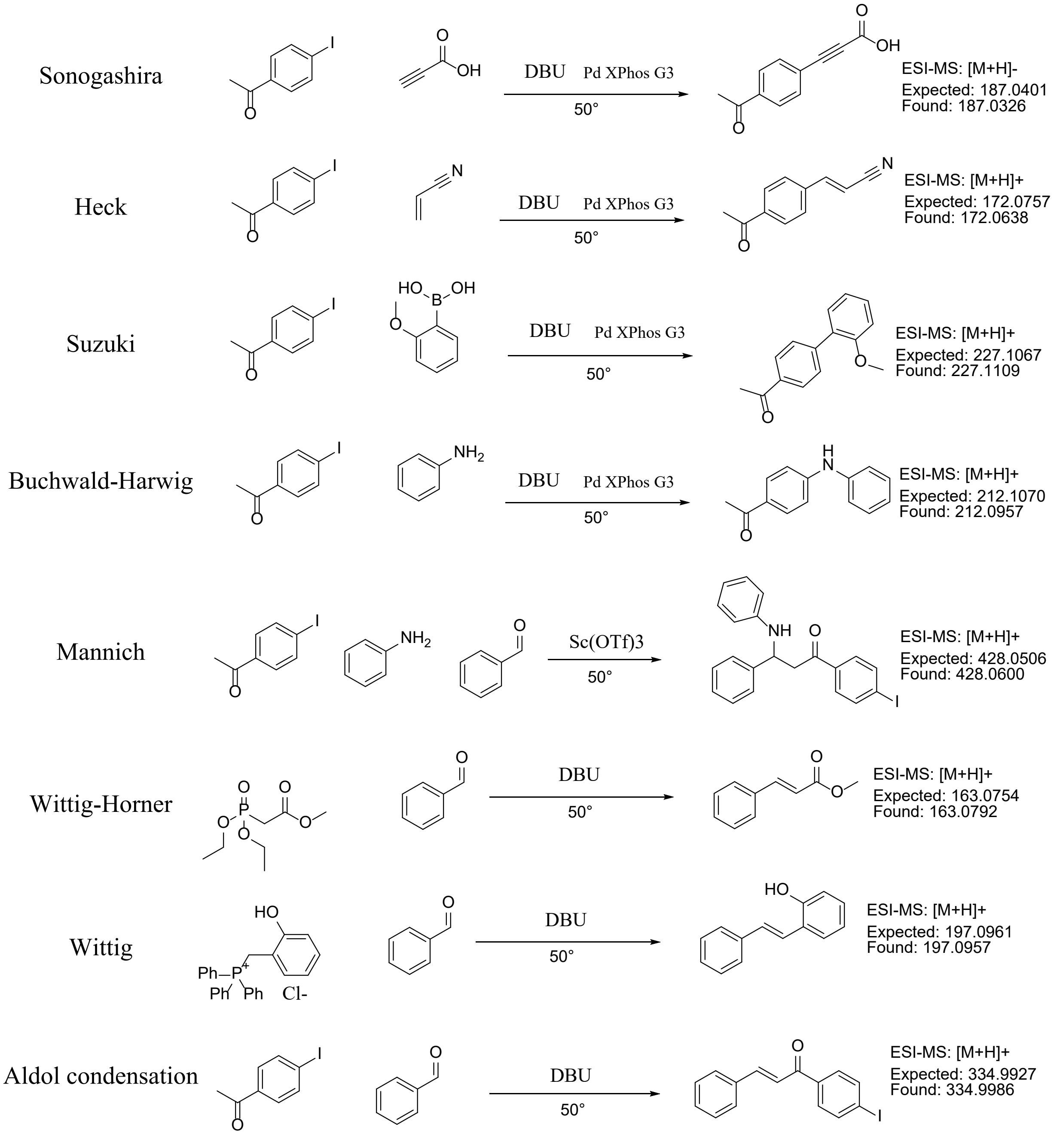

Figure S13: Named reactions in the rediscovery space were performed manually and analysed with an offline HPLC-MS system, confirming the presence of the expected products.

6. Results inferred from probabilistic model

Due to our choice of priors, the properties are initially allocated very sparingly by the system, but as more reactive combinations are encountered, the number of allocated properties rose to six by end of exploration to account for positive reactivity observations, Figure S15a,b.

By comparing the likelihood of the reactions during the exploration, it is possible to highlight analogous reactions, see Figure S15c. For example, although the Heck reaction is encountered early on, it is only established as a credible reactivity after several analogous reactions, *e.g.* Suzuki and Buchwald–Hartwig, have been observed. Reactive observations that remain unlikely even at the end of the experiment, *e.g.* the Mannich reaction, serve to point out incorrect assumptions or bias in the model, incorrect reactivity assignments, or the presence of unexpected and unknown reactivity modes that are worth exploring further. A key insight is that the most reactive combinations are not necessarily the most interesting. Specifically, examining the region of chemical space containing the reactions of historical interest (blue boundary, Figure S15d), shows that these combinations are distributed over a wide range of reactivities. In other words, using an ideal reactivity seeking artificial intelligence would not have revealed any of these reactions.

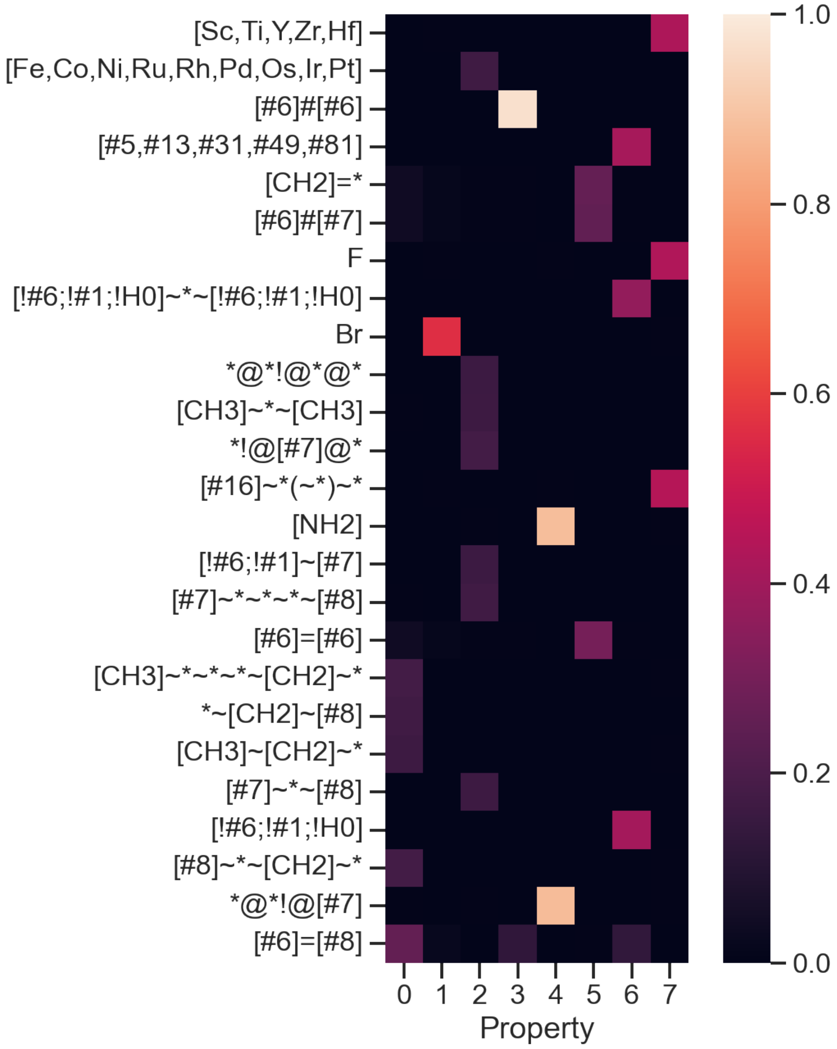

Figure S14: Exploration of a chemical space containing landmark discoveries. a) The chosen prior for properties has an explicit bias towards assigning the least possible of number properties. When observations counter to this prior belief are made, the system gradually adapts to explain them. b) Allocated properties for all compounds at the beginning (step 0) and end (step 549) of chemical space exploration. c) Likelihood values for name reactions. In line with the priors chosen, all reactive entries are initially seen to be highly unlikely. As more observations are revealed, the model is able to justify these reactive combinations. d) Projection of experiments in this chemical space onto two dimensions (via principal component analysis) overlaid on an interpolated map showing the reactivity value at each point. Darker shades indicate areas containing more reactive combinations as defined by the number of HPLC regions containing new peaks. The occurrence of neither landmark discoveries (blue) nor unexpected reactions (green) appears linked to the reactivity of a combinations (darker shaded areas), i.e., neither class of reaction is likely to have been discovered by a reactivity-seeking algorithm.

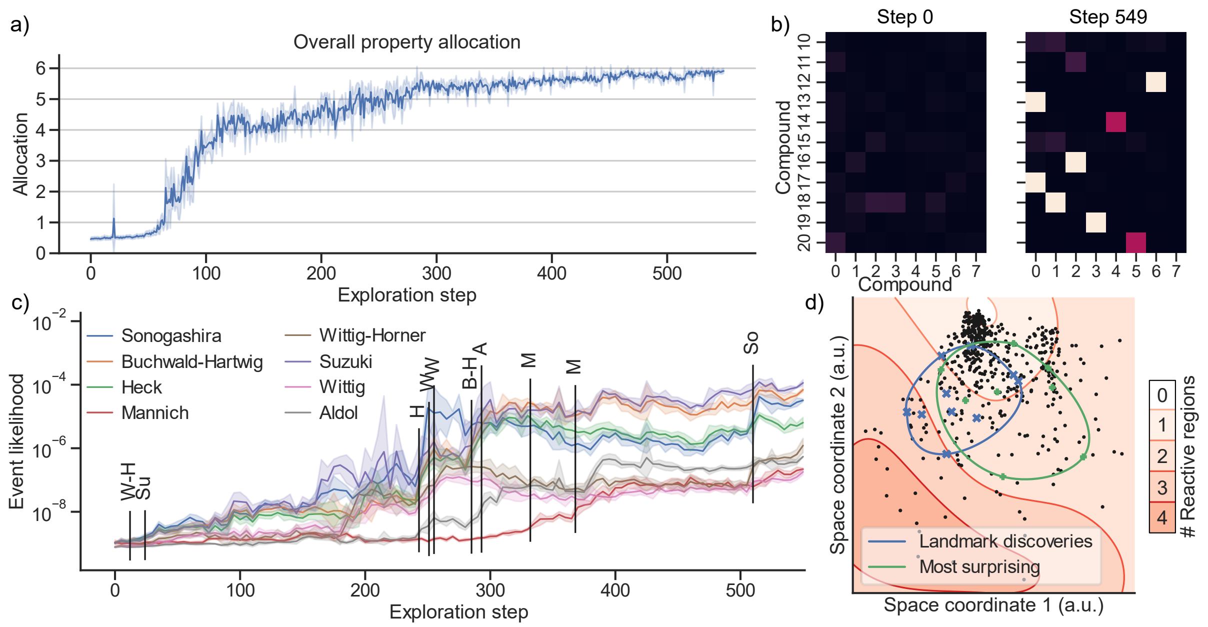

Figure S15: Exploration of a chemical space containing landmark discoveries. a) The chosen prior for properties has an explicit bias towards assigning the least possible of number properties. When observations counter to this prior belief are made, the system gradually adapts to explain them. b) Allocated properties for all compounds at the beginning (step 0) and end (step 549) of chemical space exploration. c) Likelihood values for name reactions. In line with the priors chosen, all reactive entries are initially seen to be highly unlikely. As more observations are revealed, the model is able to justify these reactive combinations. d) Projection of experiments in this chemical space onto two dimensions (via principal component analysis) overlaid on an interpolated map showing the reactivity value at each point. Darker shades indicate areas containing more reactive combinations as defined by the number of HPLC regions containing new peaks. The occurrence of neither landmark discoveries (blue) nor unexpected reactions (green) appears linked to the reactivity of a combinations (darker shaded areas), i.e., neither class of reaction is likely to have been discovered by a reactivity-seeking algorithm.

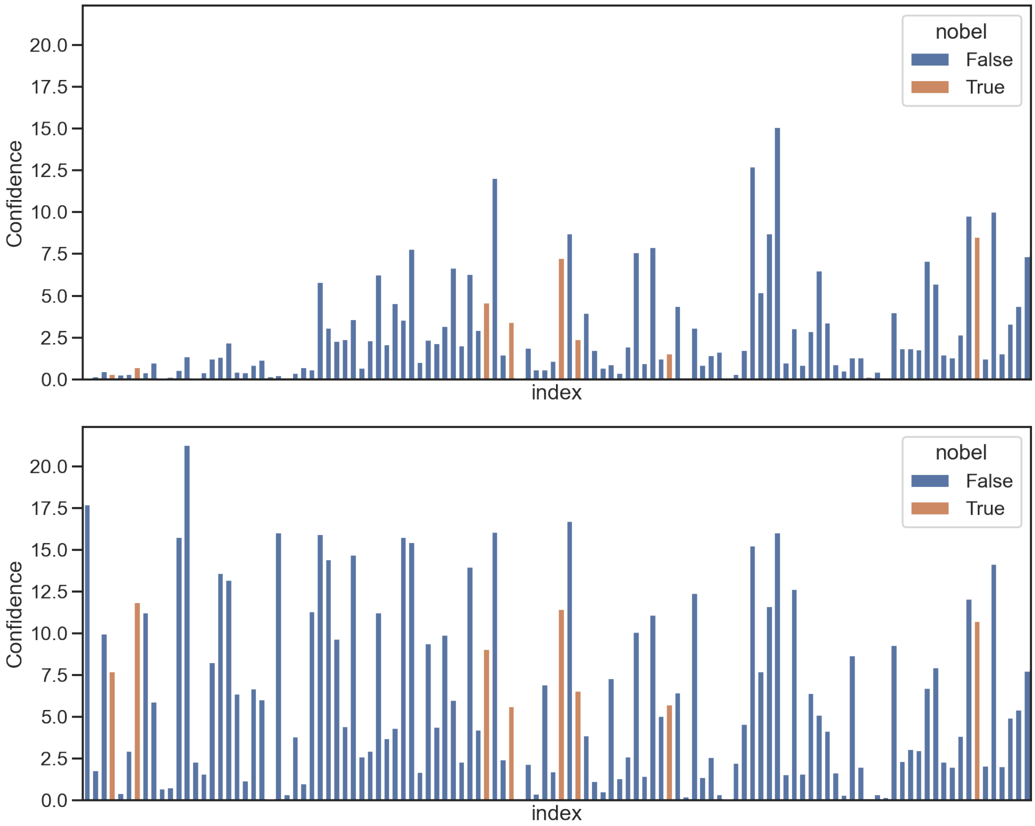

Figure S16. *A priori* (top) versus *a posteriori* (bottom) likelihood of reactions orderred according to the sequence in which they were executed.

7. References

1. Sethuraman, J. A CONSTRUCTIVE DEFINITION OF DIRICHLET PRIORS. *Statistica Sinica* **4**, 639–650 (1994).
2. Dietz, L. *Directed factor graph notation for generative models*. (2022).
3. Aggarwal, C. C. Probabilistic Graphical Models. in *Artificial Intelligence: A Textbook* (ed. Aggarwal, C. C.) 385–408 (Springer International Publishing, 2021). doi:10.1007/978-3-030-72357-6_11.
4. Phan, D., Pradhan, N. & Jankowiak, M. Composable Effects for Flexible and Accelerated Probabilistic Programming in NumPyro. *arXiv preprint arXiv:1912.11554* (2019).
5. Bingham, E. *et al.* Pyro: Deep Universal Probabilistic Programming. *J. Mach. Learn. Res.* **20**, 28:1-28:6 (2019).

6. Salvatier, J., Wiecki, T. V. & Fonnesbeck, C. Probabilistic programming in Python using PyMC3. *PeerJ Computer Science* **2**, e55 (2016).

7. Virtanen, P. *et al.* SciPy 1.0: Fundamental Algorithms for Scientific Computing in Python. *Nature Methods* **17**, 261–272 (2020).
